# Measurement of serum irisin in the different stages of chronic kidney disease

**DOI:** 10.22088/cjim.10.3.314

**Published:** 2019

**Authors:** Javad Sadeghi Shad, Roghayeh Akbari, Durdi Qujeq, Karimollah Hajian-Tilaki

**Affiliations:** 1 Cellular and Molecular Biology Research Center (CMBRC), Health Research Institute, Babol University of Medical Sciences, Babol, Iran; 2 Student Research Committee, Babol University of Medical Sciences, Babol, Iran; 3 Department of Clinical Biochemistry, Faculty of Medicine, Babol University of Medical Sciences, Babol, Iran; 4 Department of Internal Medicine, Babol University of Medical Sciences, Babol, Iran; 5 Clinical Research Development Unit of Ayatollah Rouhani Hospital, Babol University of Medical Sciences, Babol, Iran; 6 Department of Statistics and Epidemiology, School of Medicine, Babol University of Medical Sciences, Babol, Iran; 7 Social Determinants of Health Research Center, Health Research Institute, Babol University of Medical Sciences, Babol, Iran

**Keywords:** Chronic kidney disease, Irisin, Stage

## Abstract

**Background:**

Irisin is a myokine that regulates energy metabolism by inducing browning of adipose tissue. The aim of this study was to evaluate the relationship between irisin level and biochemical parameters of chronic kidney disease (CKD) patients in stage 2 and stage 4**.**

**Methods:**

The research was a cross-sectional study; the study population included patients with CKD who were over 18 years of age, included 90 individuals with CKD, of these participants, 45 were in the second stage of the CKD while the other 45 subjects were in the fourth stage. Serum irisin concentration plus the level of glucose (Glu), urea, creatinine (Cr) and hemoglobin (Hb) were measured.

**Results:**

In the present study, the serum irisin level of patients in stage 4 was significantly reduced (13.00 ng / ml) compared with patients in stage 2(21.41 ng / ml).

**Conclusion:**

With the progression of CKD from stage 2 to stage 4, parameters such as serum Cr, TG, LDL, FBS, BUN and urea levels significantly increased. Inversely, factors such as irisin, GFR, Alb, HDL and Hb levels significantly decreased. These findings suggest that irisin may be involved in the regulation of biochemical factor levels in CKD patients through the progression from stage 2 to stage 4.

Last stage of chronic kidney disease (CKD) is known as the end stage of renal disease (1). At this stage, kidney function damage was almost irreversible, and for the prevention of uremia, the patient undergoes dialysis or kidney transplantation(2, 3).Several factors contribute to the development of this disease, including hypertension, obesity, diabetes and cardiovascular disease(4). CKD as a syndrome can cause some of these disease including cardiovascular disease (CVD), hypertension and anemia (9). Several studies have been done to classify chronic kidney disease, and this classification is done when the disease has been created for three months or more based on the rate of glomerular filtration, estimated GFR (eGFR): more than 90 mL/min per 1·73 m² (stage 1 normal or high), 60–89 mL/min per 1·73 m² (stage 2 mildly decreased), 45–59 mL/min per 1·73 m² (stage 3 a mildly to moderately decreased), 30–44 mL/min per 1·73 m² (stage 3b moderately to severely decreased), 15–29 mL/min per 1·73 m² (stage 4 severely decreased), and less than 15 mL/min per 1·73 m² (stage 5 kidney failure), when GFR is less than 15 mL/min per 1·73 m² (stage 5), in this stage, the person reaches the end stage kidney disease (ESKD) (6). In this condition, the kidney is unable to maintain the normal homeostasis of the body (5-7). 

The prevalence of CKD in the world is between 7-12%, biochemical factors change with the onset of CKD, some factors, such as Alb, are a reflection of the progression of the disease, and the amount of Alb in urine output is directly related to the progression of the disease, on the other hand, factors such as proteinuria, hypertension, hyperuricemia contribute to CKD stability (8-10). Irisin is a myokine that consists of 112 amino acids and is produced by skeletal muscle tissue, peroxisome proliferator-activated receptorα (PPARα) coactivator 1α(PGC-1α)which is also induced by exercise, stimulate the expression of fibronectin type III domain containing (FNDC5) membrane section, which is further liberated by proteolytic cleavage into the blood stream, this released part is called irisin (11, 12). Studies have argued that irisin levels change in people with chronic renal disease, and this reduction of irisin has a reverse relationship with serum urea and creatinine levels. Also, changes in the irisin level have a clear effect on lipid profiles, while reduction in irisin levels leads to reduced HDL level, furthermore, irisin can be used as therapeutic agent to treat cardiovascular disease(13, 14). Decreasing irisin serum concentration simultaneously with the increasing CKD stage can predict kidney function (15). Therefore, the aim of this study was to evaluate the relationship between irisin level and biochemical parameters of CKD patients, stage 2(early CKD) and stage 4 (stage before ESKD) and its association with CKD progression. 

## Methods

The present study is a cross-sectional study of which its population included patients with CKD who were above 18 years of age, including 90 individuals with CKD. Of these participants, 45 were in the second stage of CKD while the other 45 were in the fourth stage. The study included 20 women and 25 men in the second stage of CKD (10 were diabetic patients) as well as 28 women and 17 men in stage 4 CKD (16 were diabetics).With regard to the patient's age, creatinine and gender of the patient, the MDRD equation was used to determine stage 2 (GFR=90-60) and 4(GFR=30-15). Exclusion criteria were as follows: patients with malignancy, acute inflammation, polycystic kidney disease, acute infection, as well as pregnant women. 10 ml blood sample was collected from each patient participant in fasting condition. After that, serum centrifugation and biochemical tests were performed. This study was carried out at the Kidney Transplantation Center, Shaheed Behshti Hospital, Faculty of Medicine, Babol University of Medical Sciences, Babol, Iran from September 2016 to March 2018. All subjects in the research study provided signed informed consent of the experimental protocol recommended by the our university ethics committee, and in agreement with the Helsinki Declaration. The Ethics Committee of Babol University approved the study (MUBABOL.REC.1395.215). Laboratory measurement: Serum irisin concentration was measured via the enzyme-linked immunosorbent assay (ELISA) kit (Zell BioGmbH, Germany, ZB-13253S-H9648), the intra-assay coefficient of variability (CVs) and inter-assay (CVs) reported by the manufacturer were CV<10% and CV<12%, respectively. The sensitivity of the assay was 0.09 ng/ml and the assay range of kit was 2 ng/ml - 64 ng/ml. Furthermore, in this research study, the amount of biochemical parameters such as triglyceride (TG), total cholesterol (Chol), HDL, LDL and Glu (precision of measurement at mg / dl) and Alb (precision of measurement at g / dl) in serum was measured by standard biochemical methods as previously described with some modifications (32-38). The amount of Hb was measured by cyan hematin method.

**Statistical methods and analysis: **Continuous variables for normal distribution were tested using Kolmogorov–Smirnov test, Student’s *t*-test was used to analyze the data with normal distribution (expressed as mean ± SD), for non-normal distribution, Mann-Whitney was used (expressed as median (Q_3_-Q_1_), for the evaluation of the correlation between irisin level and parametric continuous variables, Pearson’s correlation coefficient was used, and for nonparametric variables, Spearman’s correlation was used. All reported *p-*values were 2-sided, and a *p*-value of <0.05 was considered statistically significant. Statistical analysis was performed with SPSS software Version 25; in addition, the ROC statistical test was performed to determine the diagnostic accuracy of irisin, Alb and hemoglobin (Hb) as the biochemical markers to differentiate between stage 2 and stage 4 patients with CKD. The area under the curve, cut-off point, sensitivity and specificity of each biochemical factor was calculated.

## Results

The demographic characteristics of patients were shown in table 1. The data of table 2 shows that the serum level of irisin (median) in patients in stage 2 was 21.41 ng / ml and for stage 4 was 13.00 ng / ml, based on Mann Whitney's statistical test. Irisin in patients with CKD in stage 4 showed a significant decrease in comparison to stage 2(p <0.001). As shown in table 2, median of creatinine (Cr), low density lipoprotein cholesterol (LDL), FBS,TG and also means of Chol increase from stage 2 to stage 4 of CKD. 

**Table 1:** The demographic characteristics of patients

**Variable**	**Stage 2**	**Stage 4**
Male	25	17
Female	20	28
Hypertension	11	14
Diabetic	6	20
Smoking	9	11
Diuretics	8	13
Β blockers	10	15
Calcium-channel blockers	16	18
ACE inhibitors/ARBs	13	16

On the other hand, the median of glomerular filtration rate (GFR), albumin, the mean of HDL and Hb level decrease with the increasing stage of disease. The mean of normal indices as (mean ± standard deviation) as well as the abnormal indicators as median (Q3-Q1) was shown in table 2. The Mann-Whitney test for abnormal data between two groups of CKD patients in stage 2 and stage 4 of this study showed that Cr، GFR، Alb, TG and FBS is a significant difference between the two groups. The Pearson correlation test was used to determine the relationship between serum irisin and biochemical factors separately at stage 2 and stage 4. In stage 2, these patients had a negative correlation between Cr, age, FBS, BUN, LDL, Chol, urea with serum irisin. On the other hand, GFR, TG, Hb and ALb had positive correlation with serum irisin (P>0.05), and among these parameters in stage2, factors like BUN=(r=-0.309 P=0.039)، UA=(r=-0.324 P=0.030) and Alb=(r=0.334 P=0.025) have a meaningful relationship. In stage 4, factors Cr, age, HDL, BUN, LDL, Chol and UA had a negative correlation with serum irisin level. But GFR, FBS, TG, Hb and ALb factors had positive correlation with serum irisin, and among these parameters in stage 4, factors like Cr =(r=-0.380 P=0.010) ،GFR=(r=0.413 P=0.005) and Hb =(r=0.408 P=0.005) have a meaningful relationship (table 3).

**Table 2:** Mean of normal indices as (mean±SD) as well as abnormal indicators as median (Q3-Q1)

**Variable**	**Stage 2**	**Stage 4**	**P-value**
Age	45.02±14.46	63.83±14.44	P<0.001
Urea ( mg/dl)	6.55±1.08	8.18±1.68	P<0.001
BUN( mg/dl)	37.38±3.57	41.31±6.53	P<0.001
Hb( g/l)	13.41±1.62	11.4±1.3	P<0.001
Chol ( mg/dl)	171.7±43.6	180.6±47.1	P>0.05
HDL( mg/dl)	46.17±10.43	41.85±9.87	P<0.05
**Variable**	**Median( Q**_3_ **-Q**_1_ **)**	**Median(Q**_3_ **-Q**_1_ **)**	**P-value**
Irisin(ng/ml)	21.41(8.49)	13.00(6.69)	P<0.001
Cr( mg/dl)	1.1(0.20)	2.6(0.64)	P<0.001
GFR	65.95(10.91)	22.35(8.1)	P<0.001
TG( mg/dl)	117.1(67)	160(93.5)	P<0.01
LDL( mg/dl)	89(64.50)	100(48.5)	P<0.01
Alb( g/dl)	3.8(0.87)	3.2(0.55)	P<0.001
FBS( mg/dl)	101(27.50)	121(47.5)	P<0.01

Alb, Albumin ;Cr, creatinine; GFR, glomerular filtration rate; FBS, fasting blood glucose; TG ,triglyceride; Chol, cholesterol; UA ,uric acid; BUN, blood urine nitrogen; HDL, high density cholesterol; LDL, low density cholesterol ,Hb, hemoglobin.

**Table 3:** Correlation between Irisin level and other variables in stages 2 and 4

**Stage2/variables**	**Alb**	**Cr**	**GFR**	**FBS**	**TG**	**Chol**	**UA**	**BUN**	**HDL**	**LDL**	**Hb**
Irisin	r=-0.334 p=0.025	r=-0.142 P=0.353	r=0.051 p=0.741	r=-0.147 P=0.335	r=0.108 P=0.481	r=-0.022 P=0.888	r=0.324 P=0.030	r=-0.309 P=0.039	r=0.171 P=0.260	r=-0.086 P=0.575	r=0.238 P=0.115
Stage 4/variables	r=0.174 P=0.253	r=-0.380 P=0.010	r=0.413 P=0.005	r=0.026 P=0.863	r=0.234 P=0.122	r=-0.001 P=0.994	r=-0.128 P=0.332	r=-0.202 P=0.182	r=-0.241 P=0.111	r=-0.093 P=0.543	r=0.408 P=0.005

Alb, Albumin ;Cr, creatinine; GFR, glomerular filtration rate; FBS, fasting blood glucose; TG ,triglyceride; Chol, cholesterol;, UA uric acid; BUN, blood urine nitrogen; HDL, high density cholesterol; LDL, low density cholesterol, Hb, hemoglobin.

**Figure 1:**
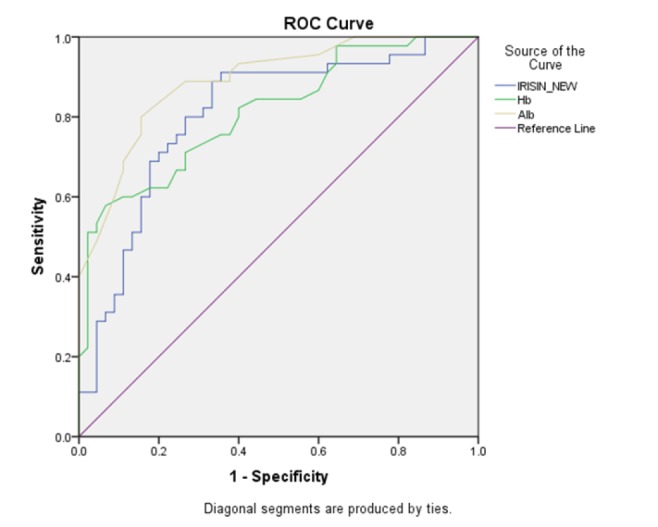
ROC curve to evaluate the sensitivity and specificity of irisin, Alb, Hb between stage 2 and stage 4

The analysis of ROC curves (figure 1) indicated that the sensitivity and specificity in the diagnosis of composite endpoint events for irisin, Alb and Hb were 80, 73% and 80, 85% and also 71, 73%, respectively. Moreover, area under the curve for irisin, Alb and Hb were 0.80, 0.81, 0.89. ROC curve to evaluate the sensitivity and specificity of irisin, Alb, Hb levels between stage 2 and stage 4 was shown in figure 1. 

## Discussion

In the present study, the serum irisin level of patients in stage 4 was significantly reduced compared with stage 2 patients, this finding is consistent with most studies that suggest that the amount of irisin is decreased in the serum of CKD patients (15, 21). One possible mechanism of the this reduction of irisin level might be due to muscle volume, because patients may have lower muscle volume in stage 4 in comparison to stage 2 . Irisin is produced within muscle, and total muscle volume can affect the irisin level. On the other hand, inflammation, oxidative stress, activation of advanced protein glycation routes, may be the causes of irisin reduction in patients with CKD (30, 31).

Moreover, study point to the fact that the reduction of irisin in subjects with CKD plays a very important role in diabetes (22). With the progression of CKD from stage 2 to stage 4, parameters such as serum Cr, TG, LDL, FBS, BUN and urea levels significantly increased. Inversely, factors such as GFR, Alb, HDL and Hb levels showed a significant reduction. These findings suggest that irisin may be involved in the regulation of biochemical factor levels in CKD patients. 

Pearson correlation analysis showed that serum irisin level in stage 2 is inversely correlated to BUN, urea levels and on the contrary irisin has direct correlation with Alb level. In stage 4, irisin is inversely associated with Cr level and directly correlated with GFR and Hb levels (24). It is also demonstrated that irisin is dependent on Hb level (13, 23-25). The results of our study are in line with the study of Ming-Shien Wen et al. on CKD patients. We observed in CKD patients that the BUN levels in stage 2 and Cr in stage 4 inversely correlated with irisin. In another study, researchers reported that with increasing irisin, HDL did not change significantly (26). 

We observed a direct correlation between serum irisin level and GFR level in stage 4 CKD patients. Irisin level decreased and was directly correlated to GFR in the advanced CKD group, and these findings confirm the results of our study (20, 26). In a systematic review and meta-analysis study, it has been shown that increased serum urea level caused increased risk of CKD progression, and urea level can be considered as a factor associated with the disease (27). In the present study, irisin had an indirect correlation with urea level in CKD stage 2. Subsequently, with the progression of CKD from stage 2 to stage 4, the amount of irisin decreased while the urea increased. This shows that irisin can be affected by urea level, however, more studies are needed in this issue. Another study has shown that there is albuminuria in CKD patients and increasing irisin significantly reduces the risk of CKD. We found that in stage 4, patients had lower serum Alb levels than stage 2, and serum Alb was directly correlated to irisin level in stage 2 (26, 28). Reducing Alb level in patients with CKD was reported (29). In the present study, the ROC curve showed that irisin has the specificity and sensitivity to differentiate between stage 2 and stage 4 in CKD.

Our study has some limitations. First, a major limitation of the study is that the concentration of other compounds was not measured in serum. This fact does not allow us to derive conclusions about other compound changes in relation progression of CKD from stage 2 to stage 4. The second limitation of our study was that present study did not allow us to infer the mechanism of action of irisin in CKD. The power of the present study is that we assessed the biochemical factors levels in CKD patients through the progression from stage 2 to stage 4 for first time.

In conclusion, the present findings suggest that irisin may be involved in the regulation of biochemical factors levels. Moreover, irisin may play a major role in affecting biochemical factors levels in CKD patients.  Lower levels of irisin in stage 4 in comparison to stage 2 are associated with lower HDL cholesterol, GFR, Hb and Alb levels. The mechanism underlying the decrease in irisin in CKD is unknown. Further basic study is needed to clarify the mechanisms underlying the effects of irisin in CKD.
